# Completeness and Consistency of Epidemiological Variables from Hospital-Based Cancer Registries in a Brazilian State

**DOI:** 10.3390/ijerph191912003

**Published:** 2022-09-22

**Authors:** Luís Carlos Lopes-Júnior, Larissa Soares Dell’Antonio, Raphael Manhaes Pessanha, Cristiano Soares Dell’Antonio, Michelaine Isabel da Silva, Thayna Mamedi de Souza, Jonathan Grassi

**Affiliations:** 1 Health Sciences Center, Nursing Department, Universidade Federal do Espírito Santo, Vitória 29043-900, Brazil; 2 Graduate Program in Public Health, Universidade Federal do Espírito Santo, Vitória 29047-105, Brazil; 3 Secretaria de Saúde do Estado do Espírito Santo, Vitória 29050-260, Brazil

**Keywords:** neoplasms, hospital-based cancer registries, quality improvement, public health surveillance, cancer epidemiology

## Abstract

Objective: To evaluate the completeness and consistency of data from hospital-based cancer registries (HCRs) in a Brazilian state. Methods: This retrospective descriptive study was based on secondary data from an HCR in the state of Espírito Santo (ES) between 2010 and 2017. The data were collected between August and November 2020 by the ES State Health Department (SESA/ES). Cancer data were obtained from the HCR of ES using the tumor registration form of the Brazilian Hospital Cancer Registry Integrator and complete databases within the SESA/ES. The incompleteness of the data was classified as excellent (<5%), good (between 5% and 10%), regular (between 10% and 20%), poor (between 20% and 50%), and very poor (>50%), according to the percentage of the absence of information. Descriptive statistical analyses were performed using Statistical Package for the Social Sciences (SPSS^®^ Inc., Chicago, IL, USA) version 20.0. Results: Complete data were observed for the variables of sex, date of the first hospital visit, and histological type of the primary tumor; that is, there were no missing data. Most epidemiological variables, including age, origin, date of first tumor diagnosis, previous diagnosis and treatment, location of the primary tumor, first treatment received at the hospital, date of death of the patient, and probable location of the primary tumor, were classified as having excellent completeness throughout the study period. However, the variables schooling, smoking, alcohol consumption, occupation, family history of cancer, and clinical staging of the tumor were classified as poor. Conclusion: Most epidemiological variables from the HCR in the state of ES, Brazil, showed excellent completeness. It is essential to elucidate the sociodemographic and clinical variables of epidemiological importance for a better understanding of the health-disease process.

## 1. Introduction

Non-communicable diseases and conditions (NCDs) are the main causes of illness and death worldwide [1]. According to the World Health Organization (WHO), NCDs lead to the death of around 41 million individuals each year, accounting for 71% of deaths worldwide [1,2], and this impact affects mainly low and middle-income countries [2]. The demographic and epidemiological transitions that occur in these countries have greatly contributed to changes in the risk profile of chronic diseases [3]. Most low- and middle-income countries continue to face high rates of NCDs, composing a scenario of a triple burden of disease, namely: (I) the unsurpassed agenda of infectious diseases and shortages, (II) the important burden attributed to deaths from external causes, and (III) the hegemonic presence of chronic conditions [4,5]. It should be noted that the profile of cancer-related risk factors has changed rapidly in developing countries, including tobacco consumption, dietary patterns, reproductive characteristics, and the prevalence of infections related to malignant neoplasms [6]. Thus, the results of the demographic, epidemiological, and nutritional transition processes worldwide signal an increasing impact of the cancer burden in the coming decades, with developing countries being responsible for the greatest global burden of cancer [7].

The latest report on the global burden of cancer in the world, using GLOBOCAN 2020 estimates of cancer incidence and mortality produced by the International Agency for Research on Cancer, with a focus on geographic variability in 20 regions of the world, estimated that there would be about 19.3 million new cases of cancer and 10 million deaths from malignant neoplasms in 2020 [6]. In both sexes, lung cancer was the most commonly diagnosed cancer (11.4% of the total) and the leading cause of cancer death (18.4% of the total cancer deaths), followed by female breast cancer (11.7%), prostate cancer (7.3%), and colorectal cancer (10%). It should be noted that the most frequently diagnosed cancer and the leading cause of cancer-related death vary substantially between and within countries depending on the degree of economic development and social and associated lifestyle factors [6].

In Brazil, according to recent data from the Brazilian National Cancer Institute (INCA), an estimated 625,000 new cases of cancer were projected to occur each year of the 2020–2022 triennium [8]. Non-melanoma skin cancer was reported to have the highest incidence (177,000), followed by cancers of the breast and prostate (66,000 each), colon and rectum (41,000), lung (30,000), and stomach cancers (21,000). The most frequent types of cancer in males, except for non-melanoma skin cancer, have been reported to be prostate (29.2%), colon and rectum (9.1%), lung (7.9%), stomach (5.9%), and oral cavity (5.0%). In females, except for non-melanoma skin cancer, breast cancer (29.7%), colon and rectum (9.2%), cervix (7.4%), lung (5.6%), and thyroid (5.4%) have been reported as the most prevalent. The distribution of incidence by geographic region shows that the Southeast region concentrates more than 60% of the incidence, followed by the Northeast (27.8%) and South (23.4%) regions [8].

The Hospital-based Cancer Registry (HCR) was developed by the National Cancer Institute (INCA) in 1980 to standardize the technical offers and training of people at a national level to improve hospital management for the care of patients with cancer. HCRs compile data on cancer cases diagnosed and/or treated at a defined institution or institution. HCRs have been developed in many lower-middle-income countries, particularly in Asia and Latin America, often because of the initiative of dedicated clinicians; they serve a range of purposes, including providing information about the diagnosis and treatment of patients in relation to specific tumor characteristics and clinical outcomes. However, depending on how the care system is organized, data on a more- or less-biased subgroup of cancer patients is collected [9]. In addition, HCRs serve as an information base for clinical-epidemiological research on the care provided to patients through the evaluation of the results of therapeutic protocols and analysis of patient survival [10,11,12,13,14,15].

Therefore, the present study aimed to evaluate the completeness and consistency of data from HCRs in a Brazilian state.

## 2. Materials and Methods

### 2.1. Study Design

This was a retrospective descriptive study based on secondary data from HCRs in the state of Espírito Santo (ES) between 2010 and 2017. The data were obtained from complete databases within the ES State Health Department (SESA/ES), as well as via download on the website of the Integrator HCR System (IHCR) of the Brazilian National Cancer Institute (INCA).

### 2.2. Ethical Issues

The project was approved by the Research Ethics Committee of the Health Sciences Center of the Federal University of ES (CEP/CCS/UFES) under protocol number 3,831,617. In addition, approval and authorization were obtained from the SESA/ES, located in the capital Vitória, for the collection of secondary data and restricted data relating to the project. It should be noted that the anonymity of the participants’ information was protected from prioritizing the principles of confidentiality and privacy related to this investigation.

### 2.3. Data Collection

Secondary data from the state of ES, located in the southeastern region of Brazil, were used. The ES Oncology Care Network covers three health regions, North/Central, Metropolitan, and South. The network comprises one High Complexity Assistance Center in Oncology (CACON), located in the municipality of Vitória, the Hospital Santa Rita de Cássia—HSRC-AFECC and seven High Complexity Oncology Care Units (UNACONs): Hospital Evangélico de Cachoeiro de Itapemirim (located in the municipality of Cachoeiro de Itapemirim), Hospital Infantil Nossa Senhora da Glória (located in Vitória), Hospital Evangélico de Vila Velha (located in the city of Vila Velha), Hospital Universitário Antônio Cassiano de Moraes (located in the capital of Vitória), Hospital Santa Casa de Misericórdia de Vitória (located in the capital of Vitória), Hospital São José (located in Colatina) and Hospital Rio Doce (located in the north of the state in Linhares). All oncology hospital units in the state have structured and functioning HCR, with their databases sent annually to the Brazilian Hospital Cancer Registry Integrator, in addition to the elaboration of the Oncology Care Line, which establishes the flow of the care network in the state of ES, with the aim of reducing mortality from neoplasms, increasing the accessibility of procedures related to the diagnosis and treatment of cancer, and improving access to health services [14,16]. Although the HCR in the state of ES, Brazil, was implemented in 2000, no assessment of the completeness of the epidemiological variables collected has been conducted, highlighting the relevance and pertinence of this study.

Data were collected between August 2020 and August 2021 at the SESA/ES. We have chosen the period 2010 to 2017 because, until December 2017, all hospitals comprising the Oncology Care Network in the state of Espírito Santo were consolidated and sent the data from the respective HCR; these were processed by the Epidemiological Surveillance of Espírito Santo state. It should be noted that data collection took place at the end of 2020 and was updated in August 2021. However, the COVID-19 pandemic imposed a great difficulty and delay on the hospitals of the Espírito Santo state in processing the sending of the HCR to the Epidemiological Surveillance for several operationalization reasons. Therefore, we decided to maintain standardization of the historical series to ensure the consolidated data from the entire Oncology Care Network of the State, i.e., considering the same period for all hospitals (CACON e UNACONs). Then, the period of 2010 to 2017 was established by the researchers for data collection.

### 2.4. Measures

To obtain data on cancer in ES, data from HCR were used. The epidemiological variables contained in the tumor registration form of the Brazilian Hospital Cancer Registry Integrator (SisRHC) [13] (Appendix A) used in this study were: (1) sex; (2) age on the date of the first consultation; (3) place of birth; (4) race/skin color; (5) schooling; (6) main occupation; (7) provenance; (8) city of residence; (9) federation unit of residence; (10) date of the first hospital visit; (11) date of first tumor diagnosis; (12) previous diagnosis and treatment; (13) most important basis for tumor diagnosis; (14) location of the primary tumor; (15) histological type of the primary tumor; (16) tumor-nodule-metastasis (TNM); (17) clinical TNM; (18) reason for not performing the treatment; (19) date of patient’s death; (20) death from cancer; (21) first treatment received in hospital; (22) disease status at the end of the first hospital treatment; (23) current marital status; (24) family history of cancer; (25) history of alcohol consumption; (26) history of tobacco consumption; (27) origin of the referral; (28) laterality of the tumor; (29) occurrence of more than one primary tumor, (30) cost of diagnosis; (31) cost of treatment.

It is noteworthy that the tumor registration form of the HCR is used to collate information from one’s medical records, provide a case summary, and as a data entry document to input information into the computerized databanks of SisRHC [13]. The content of this form is defined based on the information needs of hospitals with a hospital registry of cancer and follows the standardization guidelines suggested by the WHO through the International Agency for Research on Cancer and the Association International Cancer Registry (IACR), validated in consensus meetings coordinated by the Brazilian National Cancer Institute (INCA) [13].

### 2.5. Data Analysis

As a reference for the completeness analysis, we adopted the classification proposed by Romero and Cunha (2006) [17]. The percentage of missing data were classified as excellent (<5%), good (5–10%), regular (10–20%), poor (20–50%), or very poor (≥50%) [17,18]. The term completeness refers to the degree of completion of the analyzed field, measured by the proportion of notifications with a field filled in with a category different from those indicating the absence of data. A field filled in the database with the category “ignored”, the numeral zero, unknown date, or term indicating an absence of data was considered incomplete in this study.

The variables were analyzed according to the proportion of cases filled with non-existent codes or incorrectly formatted data. The proportion of cases of inconsistency between the following variables was also evaluated: topography and laterality, unknown primary location, and probable location of the primary tumor [13].

For the evaluation of topography and laterality, the surveillance, epidemiology, and end results inter-field editing procedures for laterality were used as a reference, considering the following valid: right, left, bilateral, and not applicable. In this sense, cases with topography that had laterality (e.g., breast, lung, kidney) were classified as inconsistent and were recorded as “not applicable”; and cases with topography that did not have laterality (e.g., trachea) and were recorded as “right”, “left” or “bilateral”. With regard to the unknown and probable locations of the primary tumor, the International Classification of Diseases for Oncology (ICD-O) and the Manual of Routines and Procedures of the HCR were used [13].

### 2.6. Statistical Analysis

Descriptive statistical analyses were performed using Statistical Package for the Social Sciences (SPSS^®^ Inc., Chicago, IL, USA) version 28.0.0 Data are presented as absolute frequencies. Based on the concept and classification of completeness of the fields [17], the tendency of non-completeness of sociodemographic and clinical variables in the HCR was analyzed. Our results have also been described using tables and graphs for further clarity.

## 3. Results

### 3.1. Frequency and Completeness of the Epidemiological Variables

The total number of cases included between 2010 and 2017 in the state of ES was 59,193 (5372, 5936, 7018, 7013, 7846, 8512, 9220, and 8276 in 2010, 2011, 2012, 2013, 2014, 2015, 2016, and 2017, respectively). Table 1 shows the frequency of cases per year from the HCR of each hospital (one CACON and seven UNACON) that is part of the Oncology Care Network of the state of ES (Table 1).

The hospital that presented better data completeness over the study period was the Hospital Santa Rita de Cássia-HSRC-AFECC for most epidemiological variables. Thirty-one variables were analyzed, of which 12 were sociodemographic (Table 2), and 19 were clinical (Table 3) variables. As this is a data completeness survey, the description of the profile of the population studied took into account the missing data. During the study period, complete data were observed for the variables of sex, date of the first hospital visit, and histological type of the primary tumor; that is, there were no missing data. Age, origin, date of the first diagnosis of the tumor, previous diagnosis and treatment, location of the primary tumor, first treatment received at the hospital, date of death of the patient, and probable location of the primary tumor were classified as having excellent completeness throughout the study period. The most prevalent classification (number of times the classification appeared, disregarding the absolute n of the variables) for both sociodemographic and clinical variables in the study period was excellent.

### 3.2. Sociodemographic Variables from the Tumor Registration form of the Brazilian HCR Integrator

The completeness of data for the sociodemographic variable place of birth in 2010 was classified as good, with 285 (5.3%) missing data; however, this variable was classified from 2011 to 2017 as regular. The schooling variable in 2010 was classified as having very poor completeness, with 2872 (53.5%) missing data. However, the schooling variable improved to a poor classification between 2011 and 2017. The variable race/color ranged between 3.5% and 14.8% of non-completeness, showing a good classification regarding the quality of the information. Regarding the occupation variable, the percentage of incompleteness ranged from 9.3% to 28.8%, with the quality of this information classified as regular. On the other hand, the marital status variable was classified as regular between 2010 and 2013, improving its classification from non-completion to good between 2014 and 2017.

The alcoholism variable showed a pattern of poor completeness, with percentages between 14.1% and 50.1%; in 2015, it was classified as very poor, with 4266 (50.1%) missing data. For the smoking variable, the percentages of non-completeness of data in the studied period were between 12.8% and 46.2%, and the completeness was classified as poor. It should be noted that the smoking variable in 2011 was classified as regular, with 759 (12.8%) missing data; however, it was then classified as poor in 2012, with 1690 (24.1%) missing data, and remained poor until 2017, with 2660 (32.1%) missing data. The variable diagnostic cost had non-completeness percentages ranging from 0.6% to 7.7% between 2010 and 2017, showing the excellent quality of information. The treatment cost variable presented parameters of completeness between 0.1% and 8.1% and was classified as good. In the same way as the diagnostic cost variable, the treatment cost variable also improved its classification in 2011, and its classification increased to excellent (0.1%); however, it returned to good in 2012, with 550 (7.8%) missing data where it remained until 2017, with 486 (5.9%) missing data (Table 2).

**Figure 1:**
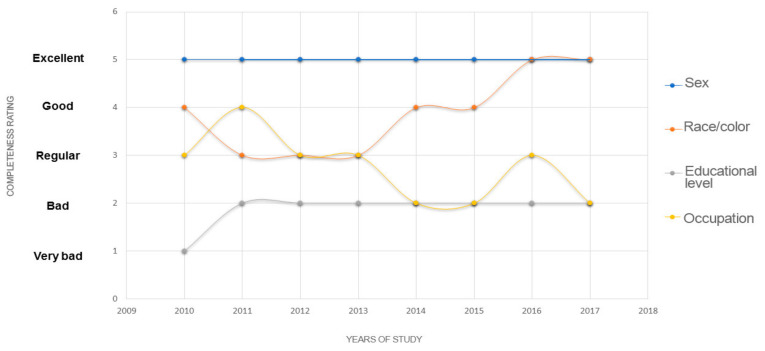
Trends in the completeness of the sociodemographic variables sex, race/color, education, and occupation in the HCR between 2010 and 2017. Vitória, ES, 2020.

Figure 1 illustrates the trends in the completeness of some relevant sociodemographic variables in the HCR, namely sex, race/color, education, and occupation, between 2010 and 2017.

### 3.3. Clinical Variables from the Tumor Registration Form of the Brazilian HCR Integrator

Regarding the clinical variables, the TNM variable was classified very poorly in all years (from 2010 to 2017), with 2012 showing the highest value of missing data, that is, 5243 (74.7%). The variable tumor staging ranged in completeness from 40.9% to 53.6% and was classified as poor. In 2012 and 2013, tumor staging had a very poor rating, with 3763 (53.6%) and 3751 (53.5%) missing data, respectively. Regarding the variable of the first specific treatment for the tumor, the percentages ranged from 1.8% to 12.5% of non-completeness; thus, the quality of the information was classified as excellent. The variable reason for not performing treatment at the hospital ranged from 4.3% to 75.4%, with the quality of information classified as regular.

The variable disease status at the end of the first treatment remained classified as poor between 2010 and 2015, rising to be classified as regular in 2016 and 2017, with 1747 (18.9%) and 1347 (16.3%) missing data, respectively. In 2010, the variable death from cancer was classified as poor, with 1254 (23.3%) missing data; however, the quality of information was improved to excellent from 2011 until 2017. In 2010, the variable referral system was classified as poor, with 1190 (22.2%) missing data, and rose to be classified as regular between 2011 and 2017. The variable family history of cancer obtained a very poor classification in all years, with percentages of non-completion between 62.5% and 70.3%; the year with the highest value of missing data was 2015, with 5980 (70.3%). The tumor laterality variable fluctuated significantly throughout the study. The tumor laterality variable was classified as regular in 2010, 2011, and 2012 with 809 (15.1%), 641 (10.8%), and 907 (12.9%) cases of missing data, respectively. In 2013, the tumor laterality variable improved to be classified as good, with 616 (8.8%) missing data. In 2014, this variable was classified as regular, with 926 (11.8%) missing data before improving to good between 2015 and 2017. With regard to the variable occurrence of more than one tumor, the percentage of non-completion ranged from 0 to 6.9% and was classified as excellent. It is noteworthy that, in 2011, zero missing data were obtained, as shown in Table 3.

**Figure 2:**
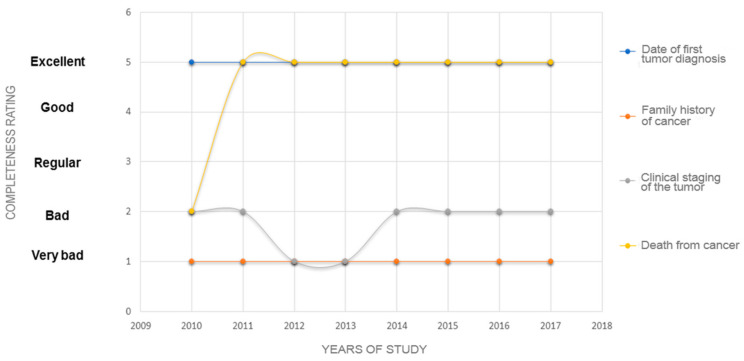
Trends in the completeness of the clinical variables: date of first tumor diagnosis, family history of cancer, clinical staging of the tumor, and death from cancer of HCR between 2010 and 2017. Vitória, ES, 2020.

Figure 2 illustrates the trends in the completeness of some clinical variables of clinical-epidemiological relevance, namely, the date of the first diagnosis of the tumor, family history of cancer, clinical staging of the tumor, and death from cancer between 2010 and 2017.

### 3.4. Inconsistency of the Epidemiological Variables

Regarding typing inconsistencies, the information “marital status”, “smoking”, “alcoholism”, and “family history of cancer” presented the same error rate of 4.49% (*n* = 2.657), that is, in the place of the correct information contained in the cancer hospital registration code, there was the code “0”, which did not exist in the tumor registration form. All of these typing errors occurred in UNACONs in the interior of the ES state (Table 4).

Of the total cancer registries, 4.49% (*n* = 5428) showed inconsistencies between the information “topography” and “tumor laterality”. Among the topographies that presented valid laterality, those that had the highest percentage of proportional filling error in relation to the total of their topography were: “ureter” (90%), “eye” (74%), “testicle” (67%), “amygdala” (62%), “adrenal gland” (59%), and “parotid gland” (55%). Additionally, among the topographies that did not have laterality, those with the highest percentages of errors were: “lips” (36%), trachea” (33%), “gallbladder” (33%), “hypopharynx” (33%), “uterus” (30%), “liver” (29%), “pancreas” (29%), “placenta” (29%) and “thymus” (29%) (Table 5).

## 4. Discussion

This review of the HCR revealed that a high number of variables were classified as excellent for completeness, especially the sex and date of the first diagnosis of the tumor. However, despite a significant number of variables classified as good or excellent, many records had incomplete information on variables of clinical-epidemiological relevance, such as education, smoking, alcohol consumption, occupation, family history of cancer, and clinical stage of the tumor disease. To better understand the health-disease process, it is essential to elucidate the sociodemographic and clinical variables of epidemiological importance [19]. In line with our findings, a study that analyzed the quality of information by verifying the completeness and consistency of the HCR from the state of Mato Grosso (MT), Brazil, found that of 15,090 cancer records obtained, the variables that were the most incomplete were TNM, schooling, the final state of the disease at the end of the first treatment, and occupation [20].

### 4.1. Sociodemographic Variables from the Tumor Registration Form of the Brazilian HCR Integrator

The sex variable was classified as excellent throughout the study period. The importance of presenting this variable with an excellent standard of completeness has been consistently reported in the literature since sex predicts the incidence of some types of neoplasms, such as breast cancer, the most common neoplasm diagnosed and the leading cause of death among women [21] and the second most commonly diagnosed cancer globally [22], thus influencing cancer estimates and clinical-epidemiological outcomes. On the other hand, prostate cancer is the second leading cause of cancer-related death among males [21,22]. A similar result was observed in a Brazilian study, in which an excellent degree of completeness was observed for sex and age; this finding may be due to the low subjectivity of the interpretation necessary to record this information [23].

It is necessary to include the race/color variable and obtain good or excellent completeness to enable data-driven public health policies to be implemented, improve services aimed at vulnerable populations in Brazil, such as blacks and browns, and resolve social and access inequalities as well as health inequities [24]. It is a complex variable that represents a set of socioeconomic conditions, the type of population, and the different conceptions of health, illness, and the problems faced, primarily by the black population [25]. Furthermore, this information is useful for debates on disqualified social inclusion and social, individual, and political programmatic vulnerability, as well as the visibility of the real need for programs, health promotion, and disease prevention in vulnerable populations [25].

The variables education and occupation are essential to collect to assess the socioeconomic conditions of individuals [26,27,28]; however, these variables were not classified as being of satisfactory completeness. Education was classified as poor in most years of the current study. Our findings corroborate some research conducted in Brazil [23,29], which showed incompleteness percentages of 69% and 31.2% for this variable. The study of the education variable is considered relevant as it can be indicative of social inequality, in addition to its usefulness as a proxy for socioeconomic factors when there is no access to income information [26,30]. The study of this variable also allows comparisons such as early diagnosis, adherence to treatment, survival assessment, and disease recurrence [30]. Several studies were carried out in an attempt to analyze the factors associated with schooling and breast cancer [31,32,33] and pointed out that, probably because of lifestyle and behavior, the results of women with higher education were better than the others. Furthermore, education has been associated with tumor size and advanced stage in the diagnosis of breast cancer [34]. Thus, it has a great impact on patient prognosis, and an improvement of its completeness is of clinical and epidemiological relevance.

Regarding the occupation variable, a study that evaluated the completeness of this information in an HCR in Brazil showed the absence of information in 45%, with a small percentage of improvement over the years of the study [35]. One study [36] described the occupational profile of individuals with leukemia and reported a data completeness percentage of 52%. In this study, a high number of sub-records of data were observed. Several studies have associated certain occupations with a greater chance of developing cancer or even dying from this cause, which emphasizes the need for greater detail in recording information related to work activities [20,37,38]. In addition to its relevance in reducing the vulnerability of patients, occupation is also an important diagnostic marker, as in cases of lung cancer, the work environment can be a place of possible exposure to carcinogens [39].

### 4.2. Clinical Variables from the Tumor Registration Form of the Brazilian HCR Integrator

The present study revealed that the clinical variable date of the first diagnosis was classified as excellent; this record is essential for defining the date of the tumor, which will be used for subsequent follow-up and calculation of indicators, particularly for the calculation of global and relative survival [13]. The variable death from cancer improved in completeness in the second year (2011) and remained excellent until 2017; this variable reflects mortality, whether due to cancer or due to another cause, and can be collected via a death certificate, progress sheet, or from another source that contains the information—an example is the collection of death certificate records of the Municipal and State Health Secretaries that are part of the Mortality Information System, of the Ministry of Health [13]. It is important that the variable death from cancer is classified as excellent in the HCR due to its clinical-epidemiological relevance and to ensure that adequate cancer surveillance can occur.

Regarding the TNM and staging variables, studies carried out in Brazil [23,29] found a “poor” degree of completeness for both variables when analyzing data from the HCR of Minas Gerais and Brazil, respectively. It is important that the TNM and staging variables are known so that the extent of the disease can be distinguished at the time of diagnosis. Based on this information, the adopted therapeutic plan is defined, making it possible to evaluate the result of the treatment administered to the person with cancer, which facilitates the standardization of procedures and exchange of experiences between institutions that offer cancer treatment. Moreover, knowing the staging also contributes to the assessment of care offered to people with cancer and helps to support the implementation of early diagnosis policies [13,40]; this information is also important because of its relevance as a prognostic factor widely used in survival studies.

Family history is a valuable marker for the early detection of cancer [41,42,43]. Furthermore, family history is associated with preventive behaviors [44]. However, the variable family history of tumors was classified as being of poor completeness across almost the entire period studied (Figure 2). Clinical staging of the tumor allows the proposed treatment to be tailored to the patient since patients with the same type of cancer, but different staging may require different therapeutic protocols [8]. Therefore, it is necessary to provide this information for personalized care in oncology [45,46].

### 4.3. Inconsistency of the Epidemiological Variables

Regarding data consistency, a considerable percentage of inconsistency between topography and laterality was observed compared to the total number of topographies contained in the bank; this may be because of the nomenclature between the topography of the ICD-O and the classification of laterality. For instance, the topography that presented inconsistent data was that referring to the colon (C 18), recorded as the ascending colon (right colon C 18.2) and descending colon (left colon C 18.6) [47]. However, in terms of laterality, the colon is a single organ [48]. It is noteworthy that in some UNACONs in the state, the registrar is not a health professional, which highlights the need for education on this subject. To improve the quality of the information in hospital databases, health professionals must have greater involvement in this work, and there needs to be an improvement in the registration of medical records. In addition, the continuous use of this information by hospital units for planning and decision-making will help to improve the quality of care provided [20,49].

### 4.4. Limitations

This study has some limitations. Although the HCR is of great value in providing a quality assessment of the services rendered, it cannot provide a clear picture of the underlying local, regional, or national epidemiology of cancer. Because the collected data are derived from either patient attendance at a given hospital or the number of cancers that have been biopsied (pathology-based systems), inclusion as a case is determined by the extent of facilities and expertise available within the respective institutions. Therefore, the aggregated cases recorded comprise a subset of the total caseload. Another limitation of this study is that the trend analysis of incomplete data was not performed using polynomial regression models.

Nevertheless, this study addresses a topic of increasing epidemiological relevance in Brazil and around the world and helps to highlight gaps in HCRs.

## 5. Conclusions

Most of the epidemiological variables reviewed from the HCR in ES, Brazil, were classified as having excellent completeness. To better understand the health-disease process, it is essential to elucidate the sociodemographic and clinical variables of epidemiological importance. The social and demographic aspects of patients with cancer are of great importance in cancer epidemiology, as such information provides us with subsidies to learn about the health-disease process.

The need for reliable and complete data collection in the HCR is evident since such registers provide data for the planning of public policies and research aimed at cancer surveillance. It is worth mentioning the importance of involving municipalities, states, and managers in reviewing the completeness of data in HCRs to enable discussions regarding improvements that can be made in terms of health information systems.

## Figures and Tables

**Table 1:** Frequency of cases per year of the HCR of each hospital in the Oncology Care Network of the state of ES. Vitória, ES, Brazil 2022 (N = 59,193 cases).

Year	Hospital/Licensed/(n) Cancer Cases							
	Hospital Santa Rita de Cássia (CACON) *	Hospital Universitário Cassiano Antônio de Moraes (UNACON) **	Hospital Evangélico de Vila Velha (UNACON)	Hospital Santa Casa de Misericórdia de Vitória (UNACON)	Hospital Evangélico de Cachoeiro de Itapemirim (UNACON)	Hospital São José (UNACON)	Hospital Rio Doce (UNACON)	Hospital Infantil Nossa Senhora da Glória (UNACON)
2010	2934	933	310	196	884	N/A	N/A	99
2011	3195	922	333	882	829	N/A	N/A	108
2012	3412	1013	481	1078	925	N/A	N/A	109
2013	3299	1397	334	954	938	N/A	N/A	91
2014	3782	1011	334	1145	967	302	195	110
2015	3489	1518	337	1286	1132	214	429	107
2016	3956	1578	324	1351	1205	310	380	116
2017	3769	1155	409	1057	941	210	283	115
Total	27,836	9587	2862	7949	7781	1036	1287	855

Abbreviations: * CACON = High Complexity Assistance Center in Oncology; ** UNACON = High Complexity Oncology Care Units; N/A = Not applicable, because that year the hospital was not yet licensed by the Ministry of Health.

**Table 2:** Absolute frequency and percentage of missing HCR data and classification of the completeness of sociodemographic variables between 2010 and 2017. Vitória, ES, Brazil.

	2010		2011		2012		2013		2014		2015		2016		2017	
Variables	*n* (%)/Classification		*n* (%)/Classification		*n* (%)/Classification		*n* (%)/Classification		*n* (%)/Classification		*n* (%)/Classification		*n* (%)/Classification		*n* (%)/Classification	
**Sex**	0	E	0	E	0	E	0	E	0	E	0	E	0	E	0	E
**Age**	1 (0)	E	52 (0.9)	E	38 (0.5)	E	17 (0.2)	E	0	E	0	E	1 (0)	E	0	E
**Birthplace**	285 (5.3)	G	599 (10.1)	R	760 (10.8)	R	702 (10)	R	1000 (12.7)	R	902 (10.6)	R	961 (10.4)	R	1048 (12.7)	R
**Race/Color**	434 (8.1)	G	801 (13.5)	R	1014 (14.4)	R	1037 (14.8)	R	642 (8.2)	G	492 (5.8)	G	389 (4.2)	E	286 (3.5)	E
**Educational level**	2872 (53.5)	VP	2261 (38.1)	P	2950 (42)	P	2826 (40.3)	P	2870 (36.6)	P	2932 (34.4)	P	3043 (33)	P	2646 (32)	P
**Occupation**	770 (14.3)	R	554 (9.3)	G	1294 (18.4)	R	1397 (19.9)	R	1736 (22.1)	P	1996 (23.4)	P	1715 (18.6)	R	2387 (28.8)	P
**Provenance**	11 (0.2)	E	52 (0.9)	E	55 (0.8)	E	30 (0.4)	E	24 (0.3)	E	53 (0.6)	E	81 (0.9)	E	29 (0.4)	E
**Marital status**	703 (13.3)	R	671 (11.3)	R	1118 (15.9)	R	1106 (15.8)	R	674 (8.6)	G	779 (9.2)	G	677 (7.3)	G	591 (7.1)	G
**Alcoholism**	1111 (20.7)	P	837 (14.1)	R	1786 (25.4)	P	2036 (29)	P	2714 (34.6)	P	4266 (50.1)	VP	4404 (47.8)	P	3072 (37.1)	G
**Smoking**	1077 (20)	P	759 (12.8)	R	1690 (24.1)	P	1948 (27.8)	P	2525 (32.2)	P	3935 (46.2)	P	3959 (42.9)	P	2660 (32.1)	G
**Cost of diagnosis**	416 (7.7)	G	36 (0.6)	E	495 (7.1)	G	344 (4.9)	E	374 (4.8)	E	421 (4.9)	E	373 (4)	E	437 (5.3)	G
**Treatment cost**	324 (6)	G	5 (0.1)	E	550 (7.8)	G	498 (7.1)	G	633 (8.1)	G	654 (7.7)	G	561 (6.1)	G	486 (5.9)	G

Abbreviations: E = Excellent, G = Good, R = Regular, P = Poor, VP = Very Poor.

**Table 3:** Absolute frequency and percentage of missing HCR data and classification of completeness of clinical variables from 2010 to 2017. Vitória, ES, Brazil. 2022.

	2010		2011		2012		2013		2014		2015		2016		2017	
Variables	*n* (%)/Classification		*n* (%)/Classification		*n* (%)/Classification		*n* (%)/Classification		*n* (%)/Classification		*n* (%)/Classification		*n* (%)/Classification		*n* (%)/Classification	
**First consultation date**	0	E	0	E	0	E	0	E	0	E	0	E	0	E	0	E
**Date of first diagnosis of the tumor**	146 (2.7)	E	107 (1.8)	E	121 (1.7)	E	9 (0.3)	E	31 (0.4)	E	163 (1.9)	E	188 (2)	E	26 (0.3)	E
**Previous diagnosis and treatment**	149 (2.8)	E	41 (0.7)	E	105 (1.5)	E	32 (0.5)	E	40 (0.5)	E	151 (1.8)	E	133 (1.4)	E	37 (0.4)	E
**Most important basis for diagnosis**	90 (1.7)	E	76 (1.3)	E	98 (1.4)	E	71 (1)	E	77 (1)	E	64 (0.8)	E	176 (1.9)	E	146 (1.8)	E
**Tumor location first**	219 (4.1)	E	217 (3.7)	E	170 (2.4)	E	142 (2)	E	179 (2.3)	E	204 (2.4)	E	188 (2)	E	166 (2)	E
**Histological type of tumor first**	0	E	0	E	0	E	0	E	0	E	0	E	0	E	0	E
**TNM**	3822 (71.1)	VP	4258 (71.7)	VP	5243 (74.7)	VP	5139 (73.3)	VP	4878 (62.2)	VP	5536 (65)	VP	5952 (64.6)	VP	5332 (64.4)	VP
**Clinical staging of the tumor**	2313 (43.1)	P	2622 (44.2)	P	3763 (53.6)	VP	3751 (53.5)	VP	3487 (44.4)	P	3859 (45.3)	P	3767 (40.9)	P	3462 (41.8)	P
**First Date specific treatment for the tumor**	674 (12.5)	R	220 (3.7)	E	377 (5.4)	G	390 (5.6)	G	219 (2.8)	E	154 (1.8)	E	225 (2.4)	E	167 (2)	E
**Reason for not performing treatment**	4052 (75.4)	VP	254 (4.3)	E	343 (4.9)	E	412 (5.9)	G	805 (10.3)	R	434 (5.1)	G	548 (5.9)	G	552 (6.7)	G
**First treatment received**	151 (2.8)	E	16 (0.3)	E	126 (1.8)	E	178 (2.5)	E	126 (1.6)	E	127 (1.5)	E	42 (0.5)	E	25 (0.3)	E
**Disease status at the end of the first treatment**	2169 (40.4)	P	2062 (34.7)	P	2291 (32.6)	P	1721 (24.5)	P	2301 (29.3)	P	2009 (23.6)	P	1747 (18.9)	R	1347 (16.3)	R
**Patient’s death date**	42 (0.8)	E	19 (0.3)	E	16 (0.2)	E	17 (0.2)	E	16 (0.2)	E	13 (0.2)	E	12 (0.1)	E	15 (0.2)	E
**Death from cancer**	1254 (23.3)	P	114 (1.9)	E	29 (0.4)	E	36 (0.5)	E	62 (0.8)	E	84 (1)	E	81 (0.9)	E	130 (1.6)	E
**Family history of cancer**	3640 (67.8)	VP	3881 (65.4)	VP	4879 (69.5)	VP	4466 (63.7)	VP	5213 (66.4)	VP	5980 (70.3)	VP	6398 (69.4)	VP	5170 (62.5)	VP
**Forwarding origin**	1190 (22.2)	P	630 (10.6)	R	1152 (16.4)	R	961 (13.7)	R	1349 (17.2)	R	1162 (13.7)	R	1234 (13.4)	R	1157 (14)	R
**Probable location of tumor first**	66 (1.2)	E	51 (0.9)	E	47 (0.7)	E	28 (0.4)	E	46 (0.6)	E	56 (0.7)	E	72 (0.8)	E	75 (0.9)	E
**Tumor laterality**	809 (15.1)	R	641 (10.8)	R	907 (12.9)	R	616 (8.8)	G	926 (11.8)	R	743 (8.7)	G	705 (7.6)	G	675 (8.2)	G
**Occurrence of more than one tumor**	311 (5.8)	G	0	E	481 (6.9)	G	334 (4.8)	E	335 (4.3)	E	383 (4.5)	E	325 (3.5)	E	414 (5)	G

Abbreviations: E = Excellent, G = Good, R = Regular, P = Poor, VP = Very Poor.

**Table 4:** Inconsistency of variables, according to the proportion of cases without information and proportion of non-existent codes, respectively, HCR/ES 2010–2017.

Variables	Incompleteness (%)	Inconsistency (%)
Sex	0.00	0.00
Age	1.60	0.00
Birthplace	10.57	0.00
Race/Color	8.60	0.00
Educational level	37.89	0.00
Occupation	20.00	0.00
Provenance	0.56	0.00
Marital status	10.67	4.49
Alcoholism	34.16	4.49
Smoking	31.34	4.49
Cost of diagnosis	5.52	0.00
Treatment cost	6.26	0.00
First consultation date	0.00	0.00
Date of first diagnosis of the tumor	13.36	0.00
Previous diagnosis and treatment	1.16	0.00
Most important basis for diagnosis	1.34	0.00
Tumor location first	2.50	0.00
Histological type of tumor first	0.00	0.00
TNM	67.84	0.00
Clinical staging of the tumor	45.65	0.00
First Date specific treatment for the tumor	4.09	0.00
Reason for not performing treatment	12.50	0.00
First treatment received	1.33	0.00
Disease status at the end of the first treatment	26.43	0.00
Patient’s death date	0.25	0.00
Death from cancer	3.02	0.00
Family history of cancer	66.94	4.49
Forwarding origin	14.92	0.00
Probable location of tumor first	0.74	0.00
Tumor laterality	10.17	9.17
Occurrence of more than one tumor	4.36	0.00

**Table 5:** Inconsistency between topography and laterality, HCR/ES 2010–2017.

Topography Encoded as a Paired Organ	n	n *	(%)
Bladder (C 67)	896	233	27
Colon (C 18)	1659	457	27
Esophagus (C 15)	1598	439	28
Gallbladder (C 23)	170	56	33
Hypopharynx (C 13)	193	62	33
Lip (C 00)	151	54	36
Liver (C 22)	320	92	29
Lower gingiva (C 03)	57	17	20
Pancreas (C 25)	456	134	29
Piriform sinus (C 12)	81	22	27
Placenta (C 58)	21	6	29
Prostate (C 61)	6565	1819	28
Rectum (C 20)	1092	300	28
Small bowel (C 17)	122	29	23
Stomach (C 16	1865	505	28
Thymus (C 37)	24	7	29
Trachea (C 33)	3	1	33
Uterus (C 55)	116	35	30
Vagina (C 52)	58	9	15
**Topography Coded As A Single Organ**	**n**	n *	**(%)**
Adrenal gland (C 74)	49	29	59
Amygdala (C 09)	169	105	62
Breast (C 50)	6671	613	9
Eye (C 69)	122	90	74
Kidney (C 64)	560	68	12
Ovary (C 56)	554	67	12
Parotid gland (C 07)	83	46	55
Testicle (C 62)	185	124	67
Ureter (C 66)	10	9	90

n = total cases for this topography in the HCR/ES database, for the period analyzed; n * = total inconsistent cases for the topography in the HCR/ES database, for the analyzed period

## Data Availability

Not applicable.
